# Acts from the cracks: Representations and positions of the decolonial in the geopolitical (de)construction of power‐entangled knowledge

**DOI:** 10.1111/bjso.12835

**Published:** 2025-01-16

**Authors:** Subas Amjad Ali, Mauro Sarrica, Gordon Sammut, Sara Bigazzi

**Affiliations:** ^1^ University of Pécs Pécs Hungary; ^2^ University of Rome Rome Italy; ^3^ University of Malta Msida Malta

**Keywords:** coloniality, community, decolonisation, epistemology, eurocentrism, geopolitics, imperialism, knowledge, social representations theory

## Abstract

This paper examines the geopolitical implications of knowledge production in psychology through two studies that respond to the growing body of work on the ‘Decolonisation of Knowledge’ and the ‘Decolonisation of Psychology’ over the past two decades. By adopting a constructivist approach, particularly through the lens of Social Representation Theory (SRT), these studies explore the ways in which geopolitical contexts shape decolonial activism within psychological and scientific discourse. The first study sheds light on the lexical divergences in the construction of knowledge within the domains of psychology. We reviewed 300 article abstracts related to decolonial studies using lexicometric analysis based on the Reinhart method (IraMuTeq). Four clusters were identified: *Educational Reform, Historical Temporalities, Social Actors,* and *Epistemological Discourse.* These clusters suggest differences in knowledge production within different geopolitical localities. The second study explores these variations by immersing itself in the perspectives and representations articulated by decolonial scholars. The second study is conducted using 12 semi‐structured interviews with academics actively engaged in decolonial efforts. The aim of the two studies is to demonstrate regional variations in decolonial discourse and highlight the ongoing influence of geopolitical factors on scientific inquiry.

## INTRODUCTION

A significant movement in thought, gaining momentum since the 1990s, advocates for decolonizing knowledge as resistance against neocolonial economic and power structures embedded in global society (Malherbe et al., 2021; Reddy & Amer, 2022). Central to this movement is the idea that knowledge holds power, often masked by claims of truth, objectivity, and neutrality. This cycle is non‐linear, as those in dominant positions control the narrative, shaping epistemologies, histories, and definitions of identity, values, and humanity—what Fanon (1963) describes as distinctions between ‘human’ and ‘half‐human.’ Psychology, deeply entwined with knowledge, plays a role in defining what is ‘normal’ and reinforces colonial power dynamics.

The movement also questions knowledge boundaries, particularly in psychology, due to the discipline's colonial roots (Malherbe et al., 2021). Psychological findings seen as ‘universal’ often fail in diverse cultural or geopolitical settings, challenging the extent and reproducibility of knowledge across contexts (Reddy & Amer, 2022). This drive for reproducibility reinforces Western frameworks, whereas a decolonial perspective rejects universality and prioritizes localized epistemologies, questioning whether universal application should remain a primary aim (Adetula et al., 2022).

Economic forces also shape knowledge production, with the term ‘Global South’ emerging from liberation movements to replace ‘Third World,’ reflecting not just poverty but colonial exploitation and neocolonial relations (Grovogu, 2011). The Global North continues to benefit from resource extraction in the Global South, with these unequal dynamics influencing global politics, trade, and knowledge production to this day (Khan et al., 2022).

Noticeable tensions and contrasts among competing geopolitical theories reflect the same questioning of the relational dimension—so the very act of dismissing the relationship makes the exploitation an invisible outcome of it. According to the dominant economic and political narrative with the withdrawal of colonial forces exploitation of the Global South ended, and nowadays economic growth is based upon meritocracy of lone countries (Hickel et al., 2022) on their responsible and accountable institutions (Moyo, 2009), on the implementation of (anti‐corruption) legal frameworks (Collier, 2007), and on adoption of liberal market‐based trade policies (Rostow, 1990). Against this innocent and *per sé* existing nation narrowed narratives, critical geopolitical voices of dependency theory (Larrain, 2013; Offiong, 1982; Santos, 1970) and world‐system theory (Goldfrank, 2000; Wallerstein, 1976) highlighted how the era of neocolonialism use the existent structure of colonial economies, with the ongoing industrial growth of the global North relying on the drainage from the Global South (Hickel et al., 2022) of both material and intellectual resources (Welch & Zhen, 2008).

In this way, it's essential to examine the geopolitical and structural dynamics at play in knowledge production. Given historical colonial influences, Western countries have often been at the forefront of driving (psychological) knowledge production. This inequality is cultivated through the use of ethnocentric research samples (Henrich et al., 2010) based on the assumption of a generalized measurable constant ‘human’, a stronger presence in editorial leadership (Arnett, 2009; Thalmayer et al., 2021), greater accessibility to funding and control over defining funding frameworks (Flint et al., 2022). Linguistic inequality further exacerbates this divide, with English serving as a gatekeeper to global academic discourse (Canagarajah, 2002). Additionally, neoliberal metrics, such as visibility and citation practices, are used to measure impact and excellence, further privileging Global North researchers (Mott & Cockayne, 2017). Compounding these issues, researchers outside the Global North face additional structural disadvantages, including insufficient material, human, and socio‐political capital (Bou Zeineddine et al., 2022) with limited access to research infrastructure and resources (Bou Zeineddine et al., 2022). The dominance of Global North‐centric research agendas (Connell, 2007) and the exclusion of local epistemologies and non‐Western knowledge systems (Bhambra, 2014) further marginalized non‐Western scholars, creating a self‐reinforcing cycle of inequality in global knowledge production.

However, it's equally important to explore emerging geographical regions that are not only contributing to reproducibility efforts, such as through ManyLabs Africa (Adetula et al., 2022), but are also leading alternative approaches to knowledge production that challenge Western paradigms. Another example is the Platforms for Social Dialogue (PDS) initiative by CLACSO (Consejo Latinoamericano de Ciencias Sociales) fosters inclusive and critical social research across Latin America, promoting alternative epistemologies.

This paper delves into the decolonisation of knowledge, with a focus on psychology. We examine both the alternative knowledge production in published articles on knowledge decolonisation (Study 1) and highlight the perspectives and representations of decolonial scholars (Study 2). While the mainstream positivist approach in psychology emphasizes objectivity—often claimed by ‘invisible researchers’—and universal principles, it implicitly assumes that humans are constant across time and culture, fragmenting their mental life into something observable and measurable. In contrast, a co‐constructivist approach—such as Social Representation Theory (SRT)—views humans from a different perspective, emphasizing the socially constructed nature of knowledge, a process in which scientists themselves are actively involved (Howarth et al., 2013). This approach considers humans, their subjectivity, and their mental processes as entangled in historical, cultural, and societal dimensions (Moscovici, 1972, 1993). This perspective is particularly important for decolonial psychology, as it addresses how psychological knowledge is shaped by colonial and neocolonial power relations. By using SRT, we seek to amplify the voices of decolonial scholars, explore their representations and positionality (Moles, 2015), and highlight how they challenge dominant frameworks, with their strong agenda of conscientization of (Freire, 2017) oppressed communities and transforming these power dynamics present also in the meaning making of psychology.

In this paper, we aim to use the Social Representations Theory (SRT) (Howarth, 2006a; Jovchelovitch, 1996; Moscovici, 1961, 1988; Wagner, 1998) as the conceptual framing to explore decolonisation activism in (psychological) science production (Study 1), and how scholars in this field challenge the existing social order in the production of knowledge contents (Study 2). Inherently, this paper is interested in seeing what positionalities emerge within the decolonial discourse.

Looking at the Global North and Global South dynamics, we argue that SRT has the potential to relate power and social practices to representational fields, to explain how knowledge, never disinterested, becomes justified and reified, as knowledge construction is always pluralistic, hybrid, ambiguous, inherently containing the possibility for tension, dialogue and conflict (Howarth, 2006a). We aim to contribute to these potential dimensions of the theory by demonstrating perspectives and lexicometric analysis of the decolonial scholarship considered in our study.

## SOCIAL REPRESENTATIONS AND KNOWLEDGE PRODUCTION

Social Representations Theory (SRT) has provided the basis for studying knowledge production starting with the work of Moscovici on Psychoanalysis (Moscovici, 1961). Knowledge construction is further explored in the conceptualisation of common sense which according to Schutz takes place interchangeably between actors in the social field (first‐degree constructions) and then inversely by scientists (second‐degree constructions) based on those everyday constructions (Natanson, 1968). In our paper, this is of implication as the exploration of the insights of decolonial scholars gives space to their viewpoints with the possibility of outlining alternative forms of knowledge production.

This idea makes room for knowledge to be understood as an intersubjective, interactionist experience and not something that exists by itself removed from one another as understood by social cognitivists (Duveen & De Rosa, 1992). In the context of mainstream and dominant forms of knowledge, this creates a third dimension of viewing how the imperialist systems are also constantly defining themselves the moment they define the Other.

Moreover, SRT emphasizes that knowledge is inherently social; it is constructed through interactions among individuals and groups within specific contexts. This perspective reveals how power dynamics operate in knowledge production, as dominant groups assert their narratives while overshadowing others. The voices of subaltern communities, particularly those from indigenous and local epistemologies, are often absent from mainstream psychological discourse, leading to a distorted understanding of human behaviour and psychological processes (Howarth, 2006a; Smith, 2012).

This, hence, also places importance on the relevance of social, political, cultural, and historical context in which social and psychological knowledge emerges. Jovchelovitch (2007) presents a nuanced analysis of the relationship between knowledge and its cultural, social, and historical circumstances. Through an interdisciplinary approach drawing from psychology, sociology, and anthropology, Jovchelovitch challenges the dominant exertion of Western conceptions of knowledge that seek a pure, context‐free understanding. Instead, she argues that representation plays a central role in shaping and understanding knowledge, linking it to individuals, communities, and cultural dimensions.

This recognition has key implications for psychology, where culturally specific theories are often presented as standardized laws. By emphasizing the contextual basis of knowledge, SRT challenges the idea of psychological universals as universally applicable across diverse populations. Duveen and De Rosa (1992) introduce *microgenesis*, a process unfolding in social interactions where individuals negotiate and resolve conflicts, shaping social identities and shared meanings in a ‘genetic moment.’ Microgenesis may prompt temporary identity shifts for particular goals, while more profound *ontogenetic* and *sociogenetic* transformations may emerge, illustrating the depth of social dialogue in shaping individual and collective representations. This framework is especially relevant to this paper's studies, which leverage decolonial perspectives to drive social transformation (Klein & Licata, 2003; Licata & Klein, 2010).

SRT—along with Moscovici's Theory of Minority Influence—enables the deconstruction of entrenched colonial narratives, facilitating critical examination and active participation of marginalized communities in the process of knowledge production. By emphasizing interaction and dialogue among diverse social groups, SRT breaks down barriers, encouraging collaboration and conflict in knowledge creation. Using SRT as an overarching framework, this paper seeks to reflect on how minority scholars within psychology construct the semantic space of the field, narrate their experiences, and address challenges in advocating for critical and political perspectives over dominant ones.

In this paper, we present two exploratory analyses of the conceptualization of decolonial knowledge in different geopolitical regions. The studies employ mixed quali‐quanti methodology. The first study analyses the growing representational field of article abstracts based on the concept of decolonisation, and the second presents expert interviews with 12 decolonial scholars in their positions on decoloniality and how they see the production of knowledge in their given geopolitical context. Finally, we will also try to contribute to the Social Representations Theory as a tool with the potential to question social order, relate power to social practices and mental attitudes (Howarth, 2006b), explain how (scientific) knowledge is never disinterested and neutral, and agency, projects, and resistance is plural, dialectical and conflictual (Bauer & Gaskell, 1999; Howarth, 2006a).

## STUDY 1

### Aim of the study

The first study aims to explore how social representations of ‘Decolonisation of Knowledge’ and ‘Decolonisation of Psychology’ appear in mainstream scientific production in English. To investigate what are the semantic universes that emerge in papers that propose a contestation of mainstream approaches. This can provide insights into the discursive framing of exploitation and development in scholarly discourse. Through a Lexicometric analysis, the study investigates the representational dimensions emerging in this discourse.

### Methodology

#### Search criteria and sample

To address our research aim, we adopted a lexicometric approach to review abstracts, allowing us to analyse lexical patterns and shifts in usage across times and contexts (Rizzoli et al., 2018). We conducted a search on Google Scholar for articles containing ‘Decolonisation of Knowledge’ or ‘Decolonisation of Psychology,’ using Google Scholar for its broad academic and non‐academic coverage, essential for accessing diverse knowledge sources beyond databases like Scopus or Web of Science. The search, conducted in June–July 2023, was limited to English‐language papers, excluding books and review chapters.

**FIGURE 1:**
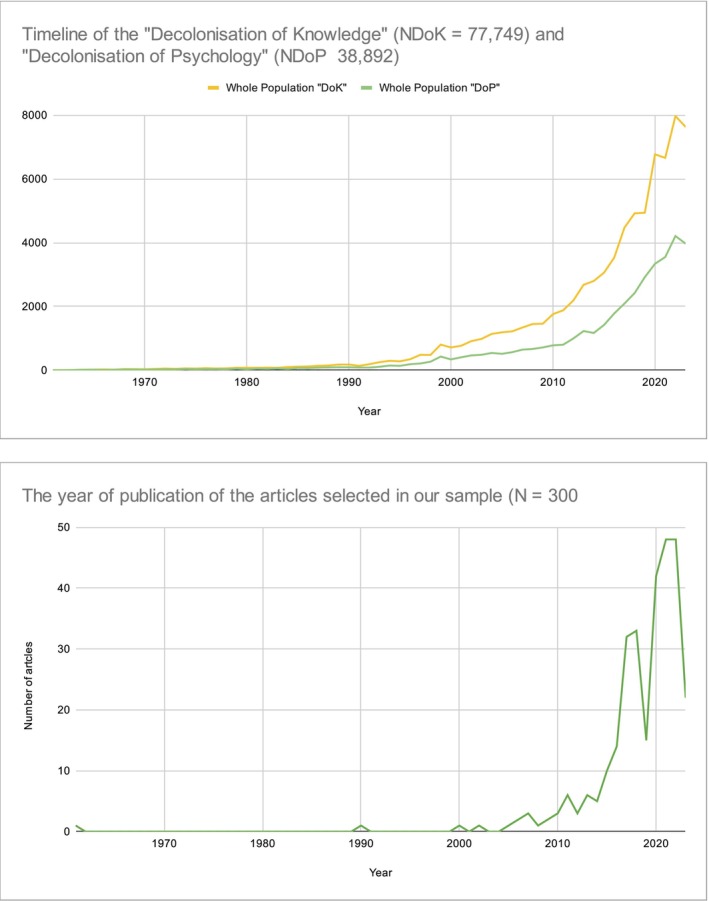
The timeline of the whole population of articles published on decolonisation between the years 1961 and 2023 and the timeline of our sample (the first 300 articles selected appearing in Google Scholar).

Our data shows a marked increase in publications on decolonisation since 2000, with entries spanning from 1961 to 2023. The search retrieved 77,749 articles on ‘Decolonisation of Knowledge’ and 38,892 on ‘Decolonisation of Psychology’ (see Figure 1). We selected the top 300 articles based on Google Scholar's ranking metrics, reflecting broader publication trends. Analysing titles and abstracts, we focused on core themes and methodologies within decolonisation, examining 53,261 words (9190 unique) to capture the complex discourse within this academic field.

For this study, a particular interest was not only to see the kind of knowledge production appearing under these search terms but also how these discussions differ regionally. In our total sample, Africa contributed 31.4% (85% of which came from South Africa contributions), Europe 24.3%, North America 23.3%, Oceania 13.7%, Asia 7.3%. The absence of articles from South America is due to the large number of texts from the region being produced in Latin languages, rather than a lack of significant contributions to the discourse. Given the predominance of South Africa emerged from the data within the African sample, we separated it out in the analysis to capture the variation in the dataset that might otherwise be obscured by its dominance. This decision was made to enable a more nuanced analysis of regional patterns across the African continent, rather than to suggest South African exceptionalism.

In a post‐truth era where facts are often contested, we separated South Africa from the broader African context to address how power dynamics shape knowledge production. While mindful of potentially reinforcing South African exceptionalism, the significant contributions from this region warranted distinct analysis to reflect regional patterns accurately. This approach also underscores the limitations of the Anglo‐normative framework in psychology, perpetuating academic elitism (Bashir et al., 2021) and reinforcing Western‐dominated knowledge systems (Adams et al., 2015).

Examining the regional disparities among the 300 articles on decolonial discourse by geopolitical locality reveals how historical power imbalances shape academic production. Formerly colonized regions often face obstacles in publishing, as they struggle to align their research with the established, imperialist scientific frameworks of the Global North. The dominance of English and stringent scientific standards further complicate this, as does the limited scientific infrastructure in many of these regions. Researchers in the Global South also manage heavy academic workloads, including teaching, project management, and administration, further reducing their capacity to contribute to international publications (Bou Zeineddine et al., 2022).

This inequality is evidenced by the dominance of articles from Europe (73 articles) and North America (70 articles), illustrating how regions with historical and economic advantages facilitate greater research output, potentially reinforcing neocolonial and hegemonic narratives (Altbach, 2013; Mosbah‐Natanson & Gingras, 2013). Oceania contributed 41 articles, Asia 22 articles, and Africa 94 articles, with 80 of those from South Africa. The absence of articles from South America can be attributed to a large number of texts being produced in Latin languages, which may not align with the English‐language bias in academic publishing. These disparities underscore the continued impact of global power dynamics on academic production, where regions with more resources, networks, and access to academic capital dominate the discourse, while marginalized regions struggle to have their voices heard.

The corpus of abstracts (*N* = 300) was analysed using IraMuTeq, a lexicometric tool developed by Pierre Ratinaud in 2009. This software, based on R and Python, provides a range of text analysis options from basic lexicography, like lemmatization and word frequency, to advanced techniques, including Descending Hierarchical Analysis (DHA) and Similarity Analysis, which are employed in this paper.

DHA clusters text segments (TS) based on vocabulary, classifying them by frequency and organizing clusters of similar but distinct vocabularies. Similarity Analysis identifies co‐occurring words within the same class, visualized as a tree, with branches indicating word relationships and communities showing clusters of similar text units. In our analysis, words with frequencies above 40 were used to create the tree, applying the Jaccard similarity index to account for both co‐occurrences and separate occurrences (a/(a + b + c)).

### Textual analysis

Our textual analysis of 300 abstracts yielded 1398 TS, with a total of 49,860 occurrences. The Hapax index was 6.45, indicating that 49.21% of the words appeared only once: out of 6533 forms, 3215 had a frequency of one. Merging similar terms (e.g. ‘university’ and ‘universities,’ ‘decolonizing’ and ‘decolonisation’) reduced the forms to 5206, with the number of hapax forms also reduced to 2510, or 48.21%.

**FIGURE 2:**
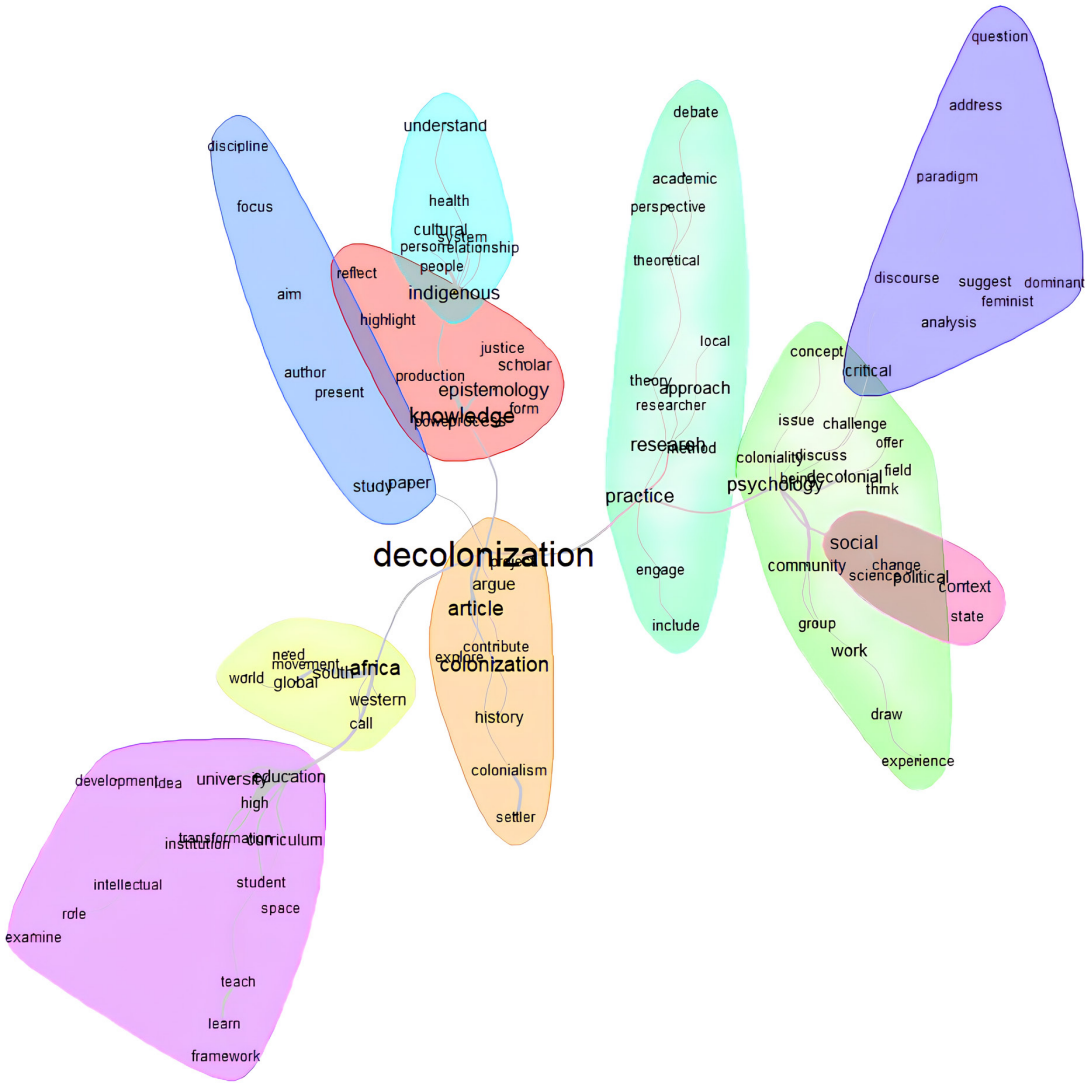
Similarity analysis of the abstracts of articles on decolonisation of knowledge and psychology.

The similarity analysis centres on ‘Decolonisation’ as the key term, reflecting its historical ties to colonization in the articles. Three main thematic halos represent the primary clusters of concepts linked to decolonisation, with a secondary halo capturing peripheral yet relevant themes, such as those related to the author's perspective (see Figure 2). This structure clarifies the distinction between central themes and contextual elements.

We categorized our articles as follows: for the Global South, we created three categories: South Africa (South Africa), Africa (Nigeria, Congo, Ghana, Algeria, Namibia), and Asia (India, Israel, Hong Kong, Pakistan, Philippines, Japan, Malaysia, Palestine, Taiwan and China). For the Global North, we established three categories: Europe (Sweden, Netherlands, Belgium, Poland, Austria, Ukraine, United Kingdom, Finland, Denmark, Germany, and Scotland), North America (Canada and the United States of America), and Oceania (Australia, New Zealand and Papua New Guinea).

### Halo 1: Africa

The first of the main halo focuses on the ***global South***, mainly South *Africa* with a need for *movement*. To this halo that of **education**, with *universities* and other *institutions*, **is** connected, calling for new *curriculum* and developing *intellectuals*.

### Halo 2: Knowledge

The second main halo is about ***knowledge** production* with new epistemologies that highlight and reflect in a way to serve justice. Another halo is connected to that of knowledge, that of ***indigenous** peoples*, *individuals*, and their *relations* which creates a *cultural system*. In this way knowledge is forged by the local, contextual, and time‐framed embodiments of social relations that from bottom to up create knowledge.

### Halo 3: Practice

The third halo is about ***practice*** strictly connected to *research*, meaning both how practice can be researched and how research can be done as a practice of decolonising, through new *methods* and *local approaches* as with new *theoretical perspectives* can a researcher contribute to the *academic debate*. To this halo, ***Psychology*** is connected. Both as *colonial* knowledge, both as a means to understand *coloniality*, or work with *communities* (Community Psychology) and *groups* creating different *experiences*, or *being decolonial* and through new ***critical*** approaches (Critical psychology) challenge the existent structure of knowledge by *analysing discourses*, by new *paradigms* addressing new *questions*, or confronting the *dominant*, *suggesting* alternative frames, such as the *feminist* one. Or act on the ***social*** (Social Psychology) to create *change* in *science* and *political contexts*.

### Specificity analysis

The Specificity Analysis of Forms examines concepts and words unique to the geopolitical localities where these articles originated. Our data (Table 1) outlines the distinct characteristics of these regions and their roles globally. However, it's important to note that many articles from the Global North are authored by individuals originally from other regions, including first‐ to third‐generation migrants from the Global South, which complicates the interpretation of regional specifics.

**TABLE 1:** Specificity analysis of forms according to the region's articles were produced.

Global south						Global north					
South Africa	*χ* ^2^	Africa	*χ* ^2^	Asia	*χ* ^2^	Europe	*χ* ^2^	North America	*χ* ^2^	Oceania	*χ* ^2^
Africa	38.70	Epistemology	6.78	Love	12.55	Psychiatry	6.28	Psychology	8.69	Indigenous	23.67
South	13.53	Africa	6.43	Indian	9.31	Academic	4.32	Food	5.94	Māori	12.23
University	10.84	Ontology	4.31	Liberal	5.97	View	3.73	Racialised	5.87	New Zeland	11.36
Education	9.51	National	4.07	Local	5.02	Think	3.72	Marginalized	5.48	Well‐being	11.09
Rationale	6.11	Political	3.85	Colonization	4.17	Geography	3.14	Therapeutic	5.29	Deprivation	9.61
Module	5.95	Experience	3.72	Colonized	3.40	British	3.04	French	5.25	Australia	9.57
Transformation	5.73	Activist	3.41	Policy	3.30	Citizenship	2.96	My	4.38	People	9.48
Decolonisation	5.36	Constitute	3.05	Confront	3.08	Human	2.75	Indigenous	4.16	Aboriginal	8.00
Protest	5.26	Day	3.04	Inclusion	2.77	Theoretical	2.67	I	3.40	Adaptation	7.03
Issue	4.36	Role	2.98	History	2.77	Address	2.65	Practice	3.82	Story	6.70
Institution	3.96	Consciousness	2.90	Academy	2.51	Former	2.59	Mental	3.73	Health	4.80
Epistemology	3.94	Appear	2.73	Active	2.30	Territory	2.41	Feminist	3.65	Settler	4.60
Curriculum	3.65	Affect	2.63	Science	2.20	Different	2.38	Community	3.53	Ally	4.51
Democracy	3.51	Associated	2.56	Language	2.10	Climate	2.37	US	2.97	Climate	4.16
Intellectual	3.09	Politics	2.56	Doing	2.01	Right	2.29	Decolonial	2.80	Relationship	3.76
Legal	3.05	Scholar	2.49			Black	2.27	Racial	2.53	Learn	3.29
Apartheid	3.03	Freedom	2.40			Dialogue	2.23	Sovereignty	2.55	Research	3.25
Special	2.58	Play	2.40			World	2.22	Anthropology	2.55	Ethical	2.96
Paper	2.33	Western	2.29			Women	2.16	Liberation	2.54	Domain	2.84
Process	2.32	Their	2.15			Potential	2.15	Live	2.52	Resource	2.71
Generate	2.24	White	2.15			Belong	2.15	Harm	2.35	Non‐indigenous	2.59
Debate	2.23	Decolonised	2.01			Author	2.14	Transnational	2.35	Project	2.54
				x		Solidarity	2.08	Researcher	2.30	Examine	2.32
	
				Race	2.08	Hegemonic	2.18	Racism	2.24
	
				Objective	2.01	Storytelling	2.14		
	
				Theory	2.01	Dutch	2.14		
	
		Colonialism	2.10						
	
		American	2.09
x	
Heal	2.09
Indigenous	−10.35	Community	−2.39	Africa	−5.18	Indigenous	−14.71	Africa	−14.73	Africa	−13.71
Settler	−6.25	Theory	−2.05	Community	−2.59	Psycology	−6.94	University	−8.94	Epistemology	−3.87
People	−3.85			Approach	−2.22	Africa	−5.68	Academic	−4.94	Decolonial	−3.18
Climate	−3.26			Student	−2.14	South	−4.68	Intellectual	−3.38	Psycology	−2.84
Essay	−2.70			Critical	−2.11	Education	−3.56	Decolonised	−3.18	Article	−2.77
Examine	−2.67					Learn	−3.47	South	−2.98	Knowledge	−2.76
Develop	−2.60					Epistemology	−3.11	Education	−2.89	South	−2.51
Relationship	−2.60					Identity	−3.00	Debate	−2.83	Debate	−2.07
Indian	−2.55					Language	−2.78	Institution	−2.57		
Critic	−2.50					Require	−2.78	Curriculum	−2.32		
Support	−2.48					Model	−2.47	Climate	−2.29		
Local	−2.44					System	−2.37	Think	−2.21		
Well‐being	−2.41					Health	−2.31				
Science	−2.30					Promote	−2.23				
Mental (health)	−2.28					Pedagogy	−2.23				
Colonialism	−2.21					Cultural	−2.20				

Verbs can also be connected to the more confrontational and active aspects of the Global South, such as *transformation*, *decolonisation*, *protest*, *experience*, *activism*, *action*, and *engagement*. Even words like *appear* and *affect* reflect more pragmatic, everyday life activities. In contrast, Global North articles tend to be more detached, often reporting scientific verbs such as *think*, *view*, *examine*, *learn*, and *research*, or discussing trauma experiences using verbs like *live*, *harm*, and *heal*, or employing abstract language to talk about everyday life, such as *dialogue*, *belong*, and *practice*. There are also differences between the subjects addressed. Specificities in the Global South pertain to themes such as *Apartheid*, *Western*, *whiteness*, *colonized*, and *decolonised*. In contrast, the Global North focuses on subjects like *settlers*, *British*, *French*, *Dutch*, and *American* on one hand, and identities like *blackness*, *indigeneity*, and *womanhood* on the other. While these terms are more specific, they can also be identified and essentialized, focusing more on labelling the social actors.

In the South African context, educational activities, universities, and curriculum transformation are under scrutiny, with other African contributions focusing on political activism. Asian articles tend to focus on reevaluating colonial history and grappling with the legacy of hegemonic and moralized relationships. European article production appears theoretical, with less emphasis on questioning epistemology and more on reporting dominated subjects such as *women* and *black* individuals, though British influence remains prominent. North American articles demonstrate a range of specificities, encompassing both outsider perspectives (*racialized, marginalized, community‐based, feminist, indigenous*) and insider views (*liberation, storytelling, lived experiences, and practices*), while also engaging in psychological aspects of domination (*mental health, harm, healing, and therapeutic approaches*). Articles from Oceania address contextual problems related to *indigenous* recognition and colonization, focusing on *co‐living relationships* and issues like *climate* change and climate justice, which are not as prominently featured in North American productions. While both American and European perspectives predominantly focus on victims and the marginalized, with less attention paid to the relationship with the dominant, Oceanian articles tend to address this aspect more comprehensively.

### Descending hierarchical analysis (DHA)

The DHA was conducted with a text corpus where the keyword ‘Decolonisation’ was removed from it, as it carried a very high frequency of occurrence (*N* = 704) and thus created a strong gravitational force around itself resulting in the rest of the corpus to become more homogenous. The reliability percentage obtained from the analysis was 91.21%, indicating a high level of confidence in the hierarchical clustering, resulting in four main classes and three clusters (Table 2).

**TABLE 2:** Summary of the descending hierarchical analysis.

N of occurrences	N. Of forms	The mean of forms by TS	N. Of forms with frequency > =3	N. Of TS	N. Of classified TS	N. Of clusters	Lemmas	N of classes
49,330	6520	35.54	1634	1388	1255	3	5145	4

In our analysis, classes represent distinct thematic clusters of keywords based on their co‐occurrence, defining different conceptual domains within the dataset. The hierarchical analysis reveals linkages between these classes, showing how themes interconnect and depend on each other. Regarding social actors, these connections highlight their multidimensional roles across clusters, illustrating the interdisciplinary nature of their involvement. For example, a social actor may be linked strongly to one class while also connecting to others, indicating their influence across various discourse domains. Hierarchical relationships clarify whether these actors are central or peripheral within themes, and how they may bridge different classes, offering insight into their roles within social, cultural, or political contexts. The analysis yielded four statistically significant classes: Class 1 (29.9%) representing Subjects or Social Actors, Class 2 (29.7%) for Epistemology, Class 3 (11.8%) for Education, and Class 4 (26.6%) for Historical Temporalities, forming three interconnected clusters (A, B, and C) as shown in Table 3.

**TABLE 3:** Descending hierarchical analysis.

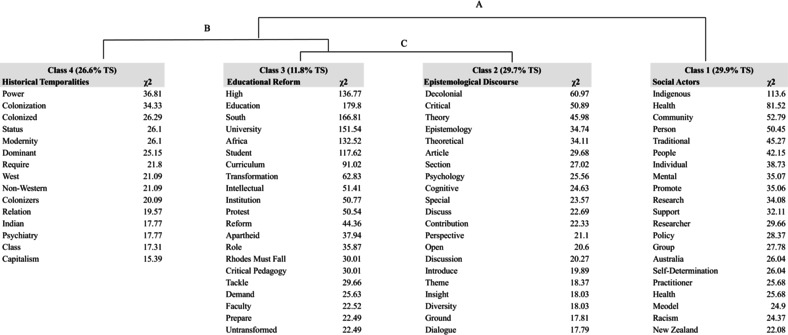

In Table 3, a summarization of the DHA is presented, highlighting the outcome of different clusters, classes, and their hierarchical relationships. Terms with a significance value of <0.0001 and with a high chi‐square value within each class are also represented, including the frequency of their occurrence in the textual corpus.

In the hierarchical analysis framework, the three clusters that emerge, illuminate the tension that exists among the identified classes and provide a relational explanation: *Educational Reform, Historical Temporalities, Social Actors*, and *Epistemological Discourse*. Working bottom‐up, Cluster C, comprising *Education* and *Epistemology*, signifies the nexus between knowledge dissemination processes and the foundational basis of knowledge construction. Drawing from geopolitical theories, *Education* serves as the conduit through which power dynamics manifest, and ideologies are taught and it further allows for the shaping of societal and often dominant narratives, while *Epistemology*, frames the epistemic boundaries and legitimizes knowledge claims within educational spheres. Cluster A centres around *Social Actors*, embodying the thematic content and disciplinary foci embedded within educational frameworks and links to the actors involved in the co‐creation of meanings. These *Social Actors* epitomize the contested terrain where hegemonic narratives intersect with subaltern voices, reflecting power struggles and geopolitical tensions within academic discourse. Lastly, the first Cluster B, *Historical Temporalities*, encompasses the social and geopolitical undercurrents and cultural influences shaping educational paradigms. Moreover, it elucidates how historical trajectories, colonial legacies, and global power dynamics permeate educational institutions, shaping pedagogical approaches and knowledge production processes. Collectively, these clusters amplify the stratified relationship between Education, Epistemology, Social Actors, and Historical Temporalities, showcasing the dynamic interactions between educational practices, knowledge frameworks, subject domains, and historical narratives, marking the multifaceted nature of educational systems within geopolitical contexts.

### Discussion

The three main thematic halos identified in the analysis—Africa, Knowledge, and Practice—shed light on the key areas of focus within the decolonial discourse. The emphasis on South Africa within the first halo highlights the country's unique historical and social conditions, which continue to shape its prominent role in the global conversation on decolonisation. The halo on Knowledge covers the centrality of epistemic justice, where indigenous knowledge systems and local cultural contexts play a pivotal role in shaping knowledge production. Meanwhile, the halo on Practice connects decolonial research with actionable steps, emphasizing the role of decolonial methodologies in reshaping academic inquiry, especially within the field of psychology.

The Specificity Analysis further illustrates how the discourse around decolonisation differs across regions, with the Global South emphasizing active transformation, protest, and engagement, while the Global North adopts a more detached, academic tone, often focusing on trauma, healing, and scientific analysis. This distinction reflects underlying geopolitical dynamics, where the Global South is often positioned in the struggle for liberation and change, while the Global North tends to approach decolonial topics through a lens of intellectual inquiry and theorization. The inclusion of regional‐specific terms like Apartheid, colonized, and whiteness in Global South articles, versus terms like settlers and British in the Global North, also points to differing historical legacies and power structures shaping decolonial thought.

The Descending Hierarchical Analysis (DHA) reveals four key thematic classes—Subjects or Social Actors, Epistemology, Education, and Historical Temporalities—that are interrelated through three clusters. These clusters highlight the tension between education and epistemology, the role of social actors in shaping decolonial discourse, and the influence of historical legacies on current knowledge systems. The geopolitical context plays a crucial role in shaping these interactions, with education serving as both a site of colonial power and a space for resistance and transformation.

These findings set the stage for a second study that will involve decolonial scholars to further explore a few key dimensions: the role of Social Actors and the intersections of Education and Epistemology in decolonial discourse identified in particular in the DHA of the abstracts.

## STUDY 2

### Aim of study

Following the Lexicometric Review, a second study was conducted to explore the temporality of the decolonial project through decolonial academics' perspectives and address questions from the abstract analysis. The interview questions examined the evolution, definitions, and future of decolonisation within today's geopolitical climate. These interviews offer insights into themes related to decolonising knowledge, particularly in framing exploitation within academic discourse. They also elaborate on epistemological consequences, and strategies of resistance and negotiation used by individuals and communities in the Global South to counter neo‐colonial exploitation. Additionally, the interviews investigate why regions like South Africa became key centers for this movement, shedding light on the historical and geopolitical shifts within the concept. Participants' reflections on decolonisation in academia, including psychology, were crucial in understanding shifts in discourse and societal awareness.

### Methodology

This study employs a qualitative analysis based on semi‐structured interviews with 12 decolonial scholars in the field of Psychology. Participants were selected through snowball sampling, with specific criteria focused on their academic engagement with Decolonial perspectives, either through previous publications or ongoing research. This sampling method allows for the inclusion of voices actively contributing to decolonial discourse.

While the sample size is small, the focus on depth and contextual insight is aligned with decolonial scholarship, which critiques traditional notions of representativeness as a construct rooted in colonial and positivist traditions. These traditions, prioritizing large sample sizes, often overlook the complexity and localized nature of knowledge in favour of generalized social realities (Roy, 2014). Instead, we follow scholars such as Walter Mignolo (2009), who emphasize the importance of situated, context‐specific knowledge, rejecting the Eurocentric bias that prioritizes universality over the richness of localized experiences.

### Procedure

The contact method began with a standardized email introducing the study, covering confidentiality, institutional affiliations, and referral information, with prior participant consent for referrals. Informed consent was obtained at each interview's start, verbally and through an online form detailing consent levels, confidentiality, and understanding. Interviews were conducted via Zoom, lasting 90–120 min, with verbal consent reviewed at each session's start. Privacy measures included data security information and the option to withdraw at any stage. Correspondence was maintained to schedule interviews.

### Participants

Twelve academics from various psychology subfields participated in the interview (*N* = 12), selected for their recognized expertise in decolonial theory and practice. Snowball sampling helped recruit participants through existing networks in the decolonial studies community. Nationalities included South African (3), American (3), Pakistani (1), British (1), Kashmiri (1), Singaporean (1), Mexican (1), and Malaysian (1), with ages from 27 to 74. Academic roles ranged from PhD student to Professor Emeritus, with career spans of 8 to over 50 years.

### Interview questions format

The questionnaire was designed based on the study of abstracts. The open‐ended questions were formulated to delve into the relationship between psychology, geopolitics, and societal transformation following temporal dimensions which divided the questions into three‐time localities: past, present, and future (Table 4).

**TABLE 4:** Questionnaire format description.

Question category	Description	Sample questions
Past (Personal & Professional Positioning)	Aims to explore participants' positioning within psychology and understanding of key historical terms like colonialism, coloniality, decolonisation, and decoloniality.	‐ ‘How do you position yourself within the field of psychology?’ ‐ ‘What does decolonisation in the field of psychology mean for you? How has this definition evolved over time, if at all?’
Present (Theoretical & Epistemological Implications)	Investigates the impact of decolonial perspectives in psychology, addressing theoretical and epistemological assumptions, methodological consequences, and the impact of decolonisation on academia and society.	‐ ‘In your experience, what are the key theoretical and epistemological assumptions or methodological consequences that arise from the decolonial perspective that challenge the mainstream field of psychology?’ ‐ ‘What is the ‘impact’ of your work?’
Future (Deconstructing Paradigms & Vision for Change)	Prompts participants to consider the need to deconstruct existing paradigms and envision new frameworks grounded in indigenous knowledge systems, as well as the future of decolonial initiatives within academia.	‐ ‘Do you find it necessary to deconstruct existing paradigms centered on colonized ideologies, or to create a separate branch based on indigenous knowledge systems? Why or why not?’ ‐ ‘What do you envision as the future of this stance in the coming 10 years?’
Self‐Reflection (Feedback & Additional Inquiry)	Encourages critical reflection on the interview questions and invites participants to suggest any additional areas of inquiry they feel are necessary, encouraging dialogue to shape the study's direction.	‐ ‘What do you think is a question that is missing or needs to be asked?’

### Interview analysis

In the analysis of the interviews, we will discuss some of the main themes, sub‐themes, and overarching themes (Table 5) which are of relevance in exploring the different decolonial knowledge dimensions related to the main themes of positionality and epistemology. The interviews were rich and deep, revealing numerous additional dimensions for exploration; however, for the purposes of this paper, we focus primarily on the above mentioned two key areas.

**TABLE 5:** Main themes, sub‐themes, and overarching themes.

Main themes	Sub‐themes	Overarching themes
Positionality	1. Advocacy for decolonisation through their academic positions	1. Activism in Academia
	2. Identification as critical psychologists	2. Transdisciplinary Collaboration
	3. Embrace of a militant academic stance	3. Social and Ethical Responsibility
	4. Differences within critical perspectives	
Education and Epistemology	1. Understanding coloniality and its dimensions	1. Re‐evaluation of knowledge production and dissemination
	2. Recognition of colonial legacy in contemporary society	2. Resistance within institutional constraints
	3. Impact of coloniality on language dynamics	3. Inclusivity and linguistic diversity
	4. Engagement with colonial mindsets and dehumanization	

### Discussion

#### Positionality

The inquiry aimed at assessing the positionality of the interviewers' answers to the question of how decolonisation is not only viewed between different academics sharing the same vein of interest but also how their own geopolitical identities locate them in this project. Several themes emerge in this domain, demanding one to see the multiplicity of this discourse.

Overall, all participants advocated for a decolonising frame, aligning themselves with the imperative to challenge colonial perspectives within their respective fields. A few participants expressed their identity as critical psychologists, signalling their intention to interrogate mainstream norms and potentially oppressive ideologies within the discipline. A term that emerged from such a position is: *‘Militant Academic’*. It spoke for a combative stance (Maldonado‐Torres, 2023) within the decolonial work, rooted from Frantz Fanon's perspective on collective responsibility (Fanon, 1963).

‘The intellectual, I am not particularly keen on the notion of intellectual. I feel more at home with the notion of a militant or someone who is a thinker or someone who is trying to think, someone who's trying to, to engage.’ (Participant N)

‘I really, really like the emphasis on combativity and in deep decolonial work there has to have a combative element. This form of decoloniality is embodied in how you position yourself, what you do outside of the university, outside of your discipline. And that work of resistance is something that goes beyond adjusting to metrics of, you know, the institutional metrics that you have. It is a lifestyle which is intrinsic to all parts of your work that goes beyond the university.’ (Participant S)

The explication of this term signals a growing recognition in academia of its activist potential to disrupt established paradigms. One participant emphasized its capacity to spark transformative changes within academic structures, allowing scholars to infuse their work with a distinct political dimension. This identity empowers researchers to approach their fields with heightened political awareness. The term's framework encourages a re‐evaluation of knowledge construction, advocating for a deconstruction of existing epistemologies. By adopting a confrontational stance toward entrenched structures, scholars are driven to challenge and dismantle prevailing norms, fostering nuanced, critical analyses. Thus, this discourse underscores the term's value as both a catalyst for academic activism and a transformative force in shaping knowledge production.

‘As Catherine Walsh says, "To create or to do your work in the cracks’, which are the interstices that are there and then try to work from there, connecting to the outside. And so, in that sense, the other category in addition to militant that I've been developing is the category of the combatant." (Participant N)

This critical approach epitomized a scholarly disposition characterized by a steadfast obligation to challenge dominant frameworks and interrogate established norms. By foregrounding their inclination to question the already existing paradigms, these scholars tried to signal a commitment to intellectual rigour but also highlighted a broader agenda for social justice within their roles as researchers.

Furthermore, the emphasis on adopting a transdisciplinary approach reflected a more compound understanding of the multifaceted nature of contemporary challenges. The scholars interviewed believed in an integrative approach that transcends disciplinary boundaries, recognizing the inherent interconnectedness of various issues and the limitations of siloed disciplinary perspectives. This openness to collaboration across diverse fields and viewpoints signified a departure from traditional academic compartmentalization, heralding a shift toward an inquiry that is layered and not purified into single components.

Integral in this stance was a recognition of the inherent complexity of present‐day issues and the inadequacy of singular disciplinary lenses in addressing them comprehensively. By embracing diverse insights and methodologies from across disciplines, scholars gestured a willingness to engage with intricacy and ambiguity, admitting that meaningful solutions often emerge from the synthesis of diverse perspectives.

In essence, this critical approach not only embodied scholars' commitment to intellectual inquiry but also served as a testament to their broader social and ethical responsibilities. By promoting and embracing transdisciplinary collaboration, these scholars aspired to cultivate a more socially responsive academic landscape capable of addressing the multipart challenges of our time.

‘I explicitly position myself as a psychologist who works very much in transdisciplinary ways. The transdisciplinary team is quite important in my work and quite central in my world.’ (Participant S)

Participants from South Africa, especially stressed the significance of centering Africa in psychology, and not psychology in Africa as a means of acknowledging the richness of African perspectives and experiences that have often been marginalized within the discipline. This kind of repositioning of not just the social actors but of the entire discipline provoked the necessity for reframing, reassessing, and ultimately rehistorising the academic scenery. As Christopher Sonn states that there is an immediate need for *‘the reconstruction of historical memory, voicing silenced stories and recognising experiences of excluded communities’* (Sonn et al., 2013 [Abstract]). This restructuring not only viewed the mainstream field with a critical lens but also promised a better one.

It is to be noted however, that while the interviewee's statement indicates their sense of belonging in a specific subfield that resonates with their values and worldview, yet, the fact that individuals may feel more aligned with fields like community psychology suggests that other areas of psychology may still reflect colonial or Western‐centric frameworks, necessitating further efforts toward decolonisation. In this sense, community psychology can be seen as a site of resistance and transformation within a broader discipline that remains, in many ways, influenced by its colonial history.

‘So, an African way of doing psychology and thinking about psychology is quite important. That is how I position myself within the discipline itself.’ (Participant S)

In the context of Psychology, two main branches were stated—community and liberation psychology. The individuals who identified with community psychology were mainly due to their extensive involvement in community situations, emphasizing collaborations with social movement actors, young people, and cultural workers, thus stressing the importance of immersion in community‐based practices. However, despite their alignment with community psychology in practice, uncertainties arose regarding the correspondence between their work and the academic representation of the field. This uncertainty reflected a broader tension between lived experiences in community practice and theoretical frameworks within academic discourse.

Moreover, a preference is expressed for liberation psychology over community psychology, citing its openness, alignment with values, and origins in anti‐apartheid movements, particularly in South Africa. Liberation psychology's emphasis on liberty and social justice resonated with these individuals. Their journey represents a complex negotiation between theory and practice, illustrating pressures and synergies between academic representations of psychology and experiences within community settings.

"The field of psychology in which I feel most at home is community psychology. Much of the work I engage in occurs within community contexts, collaborating with social movement actors, young people, and cultural workers. However, upon reading community psychology journals, I find myself thinking, 'This is interesting and commendable, but I'm uncertain if it aligns with my work.' Therefore, I find liberation psychology appealing for its openness and the absence of rigid constraints and rules." (Participant M)

This differentiation served another and more important purpose which was to express the idea of how colonization persists in the world we live in, even in abstract forms. True independence and freedom do not stem from the physical removal of a colonizer but is a process that demands self‐reflection and revaluation. For some participants, their journey therefore started with the realization that they are a product of colonization.

"Coloniality has been inside us as well. We see it everywhere outside as well…The way we live, the way we think about the world, and even our aspirations. They have been in the framework of colonialism. So, we are a product of coloniality ourselves. So, we are a product of coloniality ourselves. In the environment where I work, I encounter what we call the captive mindset. All the policies we have here, the education policies and so forth, all these things have been significantly influenced by and shaped by this colonial mindset and colonial ideas.’ (Participant F)

### Epistemology

Epistemology appears to be of an especially significant implication for the interviewees, where terms like *coloniality, decoloniality, decolonisation*, and *colonization* carried subtleties in their meanings. There was a consensual understanding of these terms however with participants offering very similar definitions to one another. For them, *coloniality* referred to the legacy of colonialism and how it continued to influence various aspects of society. One speaker explained that it operates through three axes: knowledge (how we understand the world), power (how we enact change in the world), and interactions (how we relate to each other hierarchically).

"It's in our economic relations, it's in culture, it's in the way that we just reproduce everyday life. The sort of coloniality, the legacy of colonialism, this sort of spectre of colonialism hangs over all of that, and that is sort of coloniality. The sort of three axes that they speak about operating on is knowledge, so how we know the world; power, how we make things happen in the world; and being, the way that we hierarchically relate to each other." (Participant M)

For these decolonial academics, language was an essential aspect of self‐expression and a dimension that was heavily regulated by colonial and imperial influences.

‘I think colonialism is a sociopolitical process that forced people into subjugation but in that same breath, we need to talk about how coloniality is still very much a part of our lives. So that the legacies of colonialism continue.’ (Participant G)

Others also agreed with this idea of knowledge and power being the key exploits of colonialism that persisted in the minds of people. Coloniality was analysed through its various dimensions, particularly power relations, and ways of being and knowing, therefore the knowledge systems. The discussion emphasized how coloniality seeps into the present, shaping contemporary society and influencing perceptions of what is considered ‘natural’. Psychology in this regard plays a huge role in dictating what is normal.

"Of course, psychologists always kind of knew there are other ways of being. They could read any of the anthropology books, but they always thought of those as being like a delay on a developmental trajectory or things that were going to disappear." (Participant A)

"Naming coloniality is the thing in the present, but linking it as an inherent part of modernity ties what we study in psychology and labels it as a colonial product, illuminating the colonial violence that went into producing the ways of being that we not only study but also elevate as good. We are promoting and propagating colonial ways of being." (participant G)

In defining coloniality, it tied in with their own identity as they recognized how they themselves are a product of this mechanism. They emphasized how coloniality permeates every aspect of life, shaping education, aspirations, and even authenticating processes. How the colonial mindset influenced policies, educational frameworks, and the validation of academic work, with acknowledgment and recognition often being contingent upon approval from Western scholars or publication in Western outlets.

"The captive mind or the colonial mindset entails not thinking originally, but rather adopting things from abroad and blindly following policies. So, if we try to question any policy here, if we try to say that this doesn't make sense, the only justification they have is that this is what happens in international universities as well. That's their only justification. This happens in America as well. This happens in Britain as well. So, that's taken as a justification. So, if anything is happening there, it means we don't need to question that. We don't have to question that. So, that is the framework we work in. That's our everyday living practice, and you have to resist that, even that resistance." (Participant F)

The interviewee's remarks shed light on the intricate encounters of resistance within academic settings. Despite recognizing the need to challenge colonial norms and policies, the interviewees confronted institutional barriers and pressures, such as the mandate to conduct lectures in English and the emphasis on neoliberal meritocracy‐based education policies. This highlights the tautness between institutional expectations and the desire to challenge colonial frameworks, illustrating the ongoing struggle to reconcile personal convictions with institutional constraints. Additionally, the mention of open debates in classrooms demonstrated a proactive effort to engage in critical dialogue and the need for alternative perspectives, even within these constrained environments.

"I'm officially not allowed…to question…I'm supposed to take all the lectures in English…They're very, very…the idea of improving the quality of education is having these neoliberal policies…But we do have very open debates in my class." (Participant F)

The coloniality of language is highlighted by drawing attention to the significant barricades faced by the Subaltern, especially in South Asia according to one respondent. The dominance of English in academia not only limited access to knowledge but also perpetuated the marginalization of voices expressed in local languages and dialects. This demonstrated the extent of the issue of linguistic imperialism and the erasure of diverse knowledge systems, hindering efforts to amplify marginalized perspectives and challenge colonial hegemony. Moreover, the interviewee's acknowledgment of the importance of translating and amplifying voices in local languages highlighted a commitment to inclusivity and the recognition of the inherent value of diverse linguistic and cultural expressions.

"We are trying to bring out the voices of the marginalised people, and we've tried to translate their work as well. What they do in Punjabi, what they do in their local languages, the kind of discourses they have… So, that is again, such a big audience that this discourse does not, cannot reach so many people and it's so sad. So, that also is another problem. The people, they are conversing, they are having conversations which are so important but we are not able to make good use of them. Because they're all marginalised and not given enough credit." (Participant F)

This sentiment was also shared by a scholar from South Africa who brought forth the process of dehumanization through the introduction of the role language and epistemology played in the colonial process. How this linguistic hierarchy was deeply rooted in colonial legacies, perpetuated social disparities, and shaped perceptions of identity and humanity. The concept of the ‘coloniality of language’ explained how language dynamics reflected historical power structures.

"There are 11 official languages in South Africa that people speak. Yet, you know, it's unacceptable for me to walk into a class, for example, and speak in one or two of them, creating a different dialect. Most South Africans can engage in this because they speak multiple languages, but white South Africans often only speak English or one African language and English. So, when I try to explain to students that this reflects a coloniality of language, they can see it, but it requires further explanation. It's easier to understand the practicalities, like the impact on people's lives. To speak about it as a project of rehumanization, asking what it means to be human. Often, being in the middle class, speaking English, and attending good schools are seen as markers of humanity, while others are marginalised." (Participant D)

## GENERAL CONCLUSIONS

The first study highlighted existing global inequalities in knowledge production, which likely reflect broader patterns in scientific output, even within the critical discourse of decolonisation. Significant regional disparities were evident, with South Africa dominating African contributions, while Europe and North America also contributed substantially. The absence of South American articles is largely due to the use of Latin languages, either by choice or as a barrier, making these works less accessible to English‐speaking audiences. Textual analysis in Study 1 revealed complex interactions among the need for education reforms, calls for new epistemologies emphasizing justice and indigenous perspectives, the role of social actors in contesting dominant narratives, and the recognition of historical legacies and global power dynamics. The second study focused on the positionality and epistemic proposal of decolonial scholars. Participants emphasized the complexity of this decolonial discourse, positioning themselves as critical, activist, combatant within academia with the aim to challenge dominant paradigms, highlight and question the implicit meanings perpetuating existent power and reshape from the ground, from local communities and perceivable cracks the production of new knowledges (multiplicities is an imperative here). Several themes emerged, such as transdisciplinary collaboration to address contemporary challenges, the role of language in perpetuating colonial power, the tension between theory and practice, the interrogation of mainstream norms and neoliberal political agendas. The ‘Militant Academic’ resist and take responsibility with the decolonial work, to uncover and challenge oppressive ideologies beyond academic and practice work and advocate for radical paradigm change in their disciplines. Thus, science and knowledge from this perspective is a conscious action entangled in the geopolitical fights over the symbolic fields between power positions.

Through an analysis of the differences that emerge in epistemology, the social representations of decolonial scholarship serve as an excellent illustration of the problem that academia is currently facing. Because it deals with the creation, verification, and dissemination of knowledge, this field is also vital to decolonial studies. In this framework, it serves several essential purposes. First of all, it helps to comprehend the foundations of knowledge systems, including how knowledge is produced, used, arranged, and transmitted. This understanding is necessary to analyse and critically evaluate modern knowledge systems, particularly those that have been influenced by colonialism. Secondly, it helps challenge Eurocentrism by identifying and challenging the Eurocentric biases that have shaped many traditional knowledge systems, especially in psychology, due to the historical dominance of European powers during the colonial era, thus allowing a more all‐encompassing representation of diverse worldviews. Thirdly, it offers insights into power dynamics that influence the production and dissemination of knowledge, aiding in the critique and rectification of imbalances in epistemic authority and representation caused by historical power structures. There is an emphasized need for, ‘conscious decolonisation of knowledge creation’ (Seedat & Suffla, 2017).

Epistemology within decolonial scholarship plays a key role in education, as it questions and recenters colonial frameworks, transforming curricula to develop approaches that challenge dominant knowledge structures and encourage critical thinking, reflexivity, and openness to diverse traditions. Decolonial epistemologies empower marginalized communities by validating their knowledge and experiences, enabling them to reclaim narratives and reshape educational paradigms. Through this lens, education becomes a vehicle for decolonization, promoting a more equitable and inclusive representation of knowledge, which explains the significant contributions of educational studies and pedagogy to this field.

The study also demonstrates how knowledge production was instrumentalized for geopolitical outcomes. The decolonial agenda facilitates contributions to geopolitical discourses within the divides of the Global North and Global South, amplifies also the need for methodological changes within academic spheres to not simply shift power hierarchies regulating the Global North and Global South structures but also as a way to deconstruct power (Scalbert‐Yücel & Ray, 2006), bringing focus to the relationships and not only modulating the two positions, creating further disparities (Malone & Hagman, 2002). Additionally, it explores the privileged positions that dictate the narration and portrayal of marginalized cultures and histories, a focal point of contention within decolonial studies. This movement underlines an emphasis on reforming education and curricula to address such biases and the promotion of rehistorisation. Africa is a key player in this area.

This paper emphasizes the specificities and pluralities in perspectives among decolonial academics, rather than aiming for an ‘average’ opinion. Each interview contributes to a broader mosaic of knowledge, highlighting unique insights into coloniality, identity, and power. As Linda Tuhiwai Smith (1999) argues, qualitative narratives reveal truths often hidden in quantitative data, offering critical regional insights without claiming exhaustive representation. Decolonial studies actively dismantle knowledge structures serving colonial dominance, often embedded in implicit assumptions of psychological theory (Ratele et al., 2018). SRT frames both the (de)construction of knowledge in psychology and the internal tensions within the discipline. By questioning colonial epistemologies, decolonial studies transform education, promoting critical thinking, reflexivity, and diverse epistemic perspectives. This educational approach validates marginalized knowledge, empowering communities to reclaim narratives and reshape paradigms. Findings underscore the need for active roles in social transformation, with education as a vehicle for critical engagement and rehistorizing knowledge.

This paper emphasizes the specificities and pluralities in perspectives among decolonial academics, rather than aiming for an ‘average’ opinion. Each interview contributes to a broader mosaic of knowledge, highlighting unique insights into coloniality, identity, and power. As Linda Tuhiwai Smith (1999) argues, qualitative narratives reveal truths often hidden in quantitative data, offering critical regional insights without claiming exhaustive representation. Decolonial studies actively dismantle knowledge structures serving colonial dominance, often embedded in implicit assumptions of psychological theory (Ratele, Cornell, Dlamini, Helman, Malherbe, & Titi, 2018). SRT frames both the (de)construction of knowledge in psychology and the internal tensions within the discipline. By questioning colonial epistemologies, decolonial studies transform education, promoting critical thinking, reflexivity, and diverse epistemic perspectives. This educational approach validates marginalized knowledge, empowering communities to reclaim narratives and reshape paradigms. Findings underscore the need for active roles in social transformation, with education as a vehicle for critical engagement and rehistorizing knowledge.

Global political and economic power dynamics shape not only the physical (borders, territories, products, natural and human resources), but also our social‐psychological landscape (identities, relationships, representations, values, etc.). Through heavily asymmetric power relations entangled to historical legacies that dominant positions define and exert control over the terms, concepts, standards and norms of relationing, with the interest to maintain and reinforce this asymmetry. This is often obtained through dominating the psychological landscape, the extraction of resources and the cultural and scientific construction of norms related to how to be, how to act, how to create. The decolonial perspective consciously assume an active part in this symbolic fight (Dlamini, Helman & Malherbe, 2018), questioning in itself this relation and relocating the creation of psychological landscapes to the ‘dominated localities’. challenging the very foundations of the dominant frameworks and proposing entirely new ways of thinking, acting, and creating.

We used SRT for our research which instead of radical departure, is inherently dialectical and dialogical (Markova, 2000). While decolonial scholarship advocates for a fundamental transformation of knowledge systems and power structures (Mignolo, 2009; Quijano, 2000), SRT offers a valuable theoretical approach to examine the processes of meaning‐making within specific geopolitical and academic contexts (Jovchelovitch, 2007). Although SRT is not a decolonial theory in itself, it provides valuable insights into understanding how and why colonized knowledge is reified and sustained within psychological frameworks. SRT helps illuminate the processes by which dominant knowledge systems are socially constructed, normalized, and maintained through power dynamics. In this context, decolonial discourse plays a critical role in counter‐positioning, deconstructing, and challenging these reified forms of knowledge. By focusing on how knowledge is produced, disseminated, and contested, SRT allows us to examine the mechanisms that reinforce colonized relationships within psychology. Decolonial efforts engage in a continuous negotiation within power dynamics, exploring what knowledge can be said, what can be contested, and how to create new, locally rooted knowledge systems that challenge imposed structures and aim to foster intellectual and cultural autonomy.

SRT allows us to investigate how meaning is constructed around the decolonial project and how this meaning shapes actions and responses within the discipline of psychology. In doing so, SRT serves as a starting point for understanding how these discourses emerge and develop, contributing to the broader discussion of decolonial transformation. Thus, while decolonial efforts seek structural change and the dismantling of colonial power dynamics, SRT enables us to analyse how these aspirations are represented and understood within the academic community. This helps illuminate potential pathways for how radical decolonial changes might take form.

However, the SRT theory in itself also calls for taking position, such as Moscovici stated in his early work ‘We must ask what is the aim of the scientific community. Is it to support or to criticise *the social order? Is it to consolidate it or transform it?’* (1972:23), thus in any case there is a side, explicitly voiced or implicitly silenced, as the position of neutrality is all the time strengthen the dominant narrative. This citation was also referenced by Howarth in her paper *Social Representation is Not a Quiet Thing* (2006), explaining how the theory has the potential and the *‘conceptual tools to criticise the social order’* that should apply in three key domains: explicitly connecting social practices with psychological processes; understanding and voicing the reification and legitimization of different knowledge systems; and highlight the agency and resistance in the co‐construction (of identities) (Howarth, 2006a: 72). According to the object of our study, decolonial movement in psychology frames these areas. The SRT approach used in this study allows for the consideration of how this meaning‐making is developing and what radical transformation is proposed. Decoloniality is a concept entangling psychological processes to social practices, struggle for the reification and legitimation of local knowledges, and in terms of agency and resistance position themselves as ‘militants’ in academia. From the perspective of SRT, we must view social representations not as static or uniform, but as dynamic, multi‐layered constructs that evolve within a landscape of conflicting and often contrasting interests (Buhagiar & Sammut, 2020). When applying this to a project, especially one aimed at change—whether incremental or radical—it is crucial to recognize that such transformations are inherently conflictual. Social change, particularly of a radical nature, is marked by competing agendas, as diverse groups with differing power positions negotiate and contest meaning (Buhagiar & Sammut, 2020). The process is not linear nor harmonious; instead, it reflects a dialectical and dialogical tension between alternatives. Transformation, under this view, is never a smooth or quiet transition but rather a turbulent process marked by tensions between the dominant order and alternatives. While discrediting and neglecting one of the positions is a strategy used by both sides, it is effective only for the dominant position, which holds societal consensus. According to the theory of minority influence, psychological—and later social—change can only occur through dialectical dialogue and negotiation.

Because we entered into dialogue with our object of study—the decolonial movement—we found it essential to reflect on our own position in relation to it. As scholars working within the framework of SRT, we recognize that our position as allies to the decolonial movement comes with inherent challenges. SRT, while offering valuable tools for understanding how social meanings are negotiated and contested, is not immune to the critiques of perpetuating colonial legacies. We understand that despite our efforts to support decolonial aims, our research may still be viewed as complicit in the colonial discourse.

Rather than defending our theoretical approach as neutral, we acknowledge that any framework operating within the academic mainstream risks reinforcing the very power structures it seeks to critique. To address this, we position ourselves with humility, acknowledging the limits of our perspectives and actively engaging in dialogue with decolonial voices. Our goal is not only to support but to rethink the role of SRT in dismantling dominant knowledge systems by foregrounding local epistemologies and challenging the colonial foundations of psychology and knowledge production. In this sense, we recognize that meaningful transformation requires a critical reorientation of our theoretical tools and a commitment to amplifying the voices of those historically marginalized in academic discourse.

The findings of this study highlight the essential role of scientists in shaping social representations and contributing to societal change, especially within the framework of decolonial psychology. Social representations are not neutral reflections of reality; they are deeply entangled within societal structures and power relations. Scientists, through their research choices and interpretations, actively participate in the construction of these representations and play a crucial role in either maintaining or challenging existing social orders. In the context of decolonial discourse, this responsibility is heightened. Scientists must critically engage with how their work may perpetuate or dismantle colonial and neocolonial power structures. This study not only explores how decolonial discourse is represented in psychology but also calls for a broader reflection on the ethical responsibility of researchers to contribute to the transformation of these structures and promote epistemic justice.

### Limitations and future research

Many constraints that could have affected how the results were interpreted were found during this study. First, there was a chance of publication bias, which could lead to an overestimation of the effect size because studies with favourable or statistically significant results were more likely to be published.

Production from the Global North, especially in Europe and North America, shows higher specificity in chosen topics, as seen in Table 1. While these regions often focus on health sciences (e.g. psychiatry, psychology) and indigenous studies (anthropology, geography), these areas do not fully define their scholarly output. Similarly, the Global South shows specificities, with African regions focusing on political and educational sciences and Asian regions on historical frameworks, influenced by post‐colonial perspectives. However, these specificities represent aspects of their output rather than defining characteristics.

This study's use of Google Scholar offers broad access but challenges replicability due to algorithm variability and transparency issues. While documenting keywords aids consistency, unpredictability persists. In contrast, Scopus and Web of Science offer stable indexing but limit Global South representation. A multi‐database approach could enhance dataset robustness.

The paper also faced the inherent challenge of incorporating the colonizer's perspective in understanding decolonisation. Decolonial thought often critiques the binary oppositions and hierarchical structures imposed by colonial powers, such as civilized/savage, modern/primitive, or colonizer/colonized, which were historically used to justify colonial domination. This raises the concern of how much power dynamics might be unconsciously reproduced instead of decentred within the analysis. The Other will always exist, even if only to be denounced or erased, as the act of removing the Other ultimately transforms it into an object that persists in discourse. To address this, the perspective taken here aims to exist within a complex web of relations and representations (pluriversality) rather than through dichotomous comparisons. This relationality emphasizes understanding identities as being formed in relation to others without the antagonistic underpinnings associated with colonial comparisons. This paper focuses on decolonial studies and experts, with a deliberate effort in the data collection process to include primarily decolonial perspectives. However, the challenge of fully escaping colonial frameworks remains a significant limitation, necessitating ongoing critical reflection in future research. It is to also clarify here that this is indeed not a decolonial paper but the authors take deep appreciation for the project.

Moreover, biases related to language and publication dates, combined with the possibility of not having access to unpublished or grey literature, might have restricted the review's inclusivity. Despite these limitations, efforts were made to transparently present the findings and acknowledge potential sources of bias, enhancing the credibility of this lexicometric review and providing avenues for future research to address these limitations.

Future studies could explore whether thematic classes like Social Actors, Epistemology, and Education are period‐dependent, offering insights into how historical, social, and economic shifts shape decolonial discourse. For instance, comparing the 1960s' postcolonial movements to contemporary debates might reveal changes or continuities. While this study did not focus on temporal analysis, a longitudinal approach could highlight trends and clarify if decolonial discourse has evolved with global conditions.

## AUTHOR CONTRIBUTIONS

**Subas Amjad Ali:** Writing – original draft; writing – review and editing; investigation; conceptualization; methodology; data curation. **Mauro Sarrica:** Supervision; conceptualization. **Gordon Sammut:** Supervision; conceptualization. **Sara Bigazzi:** Supervision; conceptualization; methodology; writing – original draft; investigation; data curation.

## CONFLICTS OF INTEREST

The authors declare no conflicts of interest.

## Data Availability

The authors confirm that the data used in this paper is available on the OSF platform with the DOI: 10.17605/OSF.IO/2ZGD5.
